# Bioprosthetic interstrut distance subtending the preserved anterior mitral leaflet mitigates left ventricular outflow tract obstruction

**DOI:** 10.1016/j.xjon.2021.05.005

**Published:** 2021-05-21

**Authors:** Laurencie Brunel, Zoe A. Williams, Niek J. Beijerink, Benjamin M. Robinson, Innes K. Wise, Hugh S. Paterson, Paul G. Bannon

**Affiliations:** a School of Veterinary Sciences, Faculty of Sciences, The University of Sydney, Sydney, NSW, Australia; b DVC Research Portfolio, The University of Sydney, Sydney, NSW, Australia; c Institute of Academic Surgery, Royal Prince Alfred Hospital, Sydney, NSW, Australia; d Department of Laboratory Animal Services, The University of Sydney, Sydney, NSW, Australia; e Central Clinical School, Faculty of Medicine and Health, The University of Sydney, Sydney, NSW, Australia

**Keywords:** animal model, mitral valve replacement, left ventricular outflow tract obstruction, bioprosthetic valve, left ventricular contractility, AML, anterior mitral leaflet, CI, contractility index, CPB, cardiopulmonary bypass, LV, left ventricle/ventricular, LVOT, left ventricular outflow tract, LVOTO, left ventricular outflow tract obstruction, SAM, systolic anterior motion

## Abstract

**Background:**

The anterior mitral leaflet (AML) contributes to left ventricular (LV) function but is normally excised at the time of a bioprosthetic valve insertion. This study aimed to investigate methods of safely retaining the AML at the time of mitral valve replacement.

**Methods:**

Five adult sheep (57 ± 3.8 kg) each underwent 3 insertions of a bioprosthetic mitral valve (asymmetric interstrut sectors) alternating the wide and narrow interstrut distance under the AML. Each insertion was performed on normothermic beating-heart cardiopulmonary bypass, with full retention of the native valve. After each valve insertion, continuous measurements of LV and aortic pressures were recorded with echocardiographic assessment of mitral valve function. If LV outflow tract obstruction (LVOTO) was not seen on the resumption of normal cardiac output, a bolus of adrenaline was given to precipitate it.

**Results:**

Thirteen of 15 valve insertions resulted in LVOTO caused by systolic anterior motion (SAM), independent of valve orientation. The wide interstrut distance subtending the AML was associated with a greater requirement for inotropic stress to precipitate an obstruction and was associated with late systolic rather than holosystolic obstruction.

**Conclusions:**

The predisposition to and nature of LVOTO due to SAM were associated with the bioprosthetic valve interstrut distance subtending the fully retained AML and may explain the survival differences in such patients. This model represents an effective method for research into prevention of LVOTO following mitral valve replacement with preservation of the native valve.

## Graphical abstract

Schematic representation of a normothermic beating-heart ovine model for the research into prevention of left ventricular outflow tract obstruction (LVOTO) using a bioprosthetic mitral valve, with full retention of the anterior mitral leaflet (AML). All episodes of LVOTO were due to systolic anterior motion. The wide interstrut distance subtending the AML was associated with a greater requirement for inotropic stress to precipitate an obstruction and was associated with late systolic rather than holosystolic obstruction.

**Figure:**
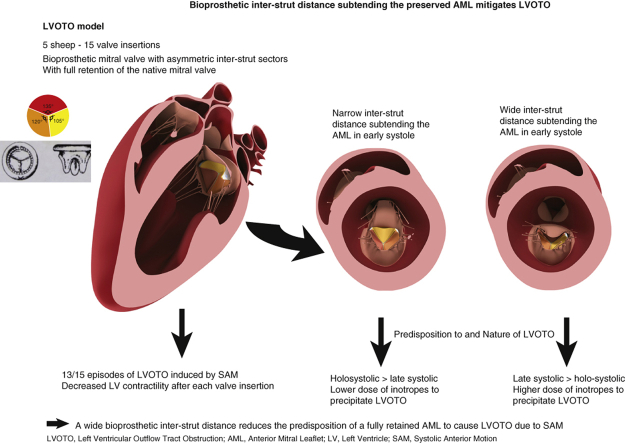

**Figure:**
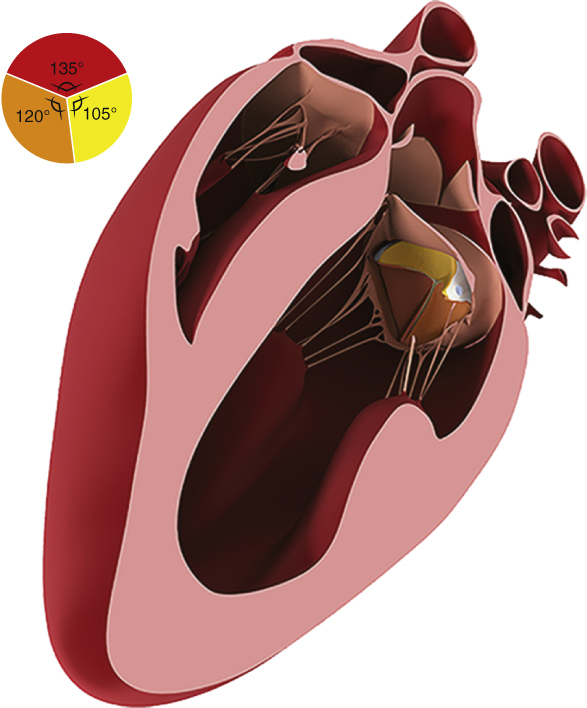
Model for research into the prevention of left ventricular outflow tract obstruction.

**Central Message**

A wide bioprosthetic valve interstrut distance reduces the predisposition of a fully retained anterior mitral leaflet to cause outflow tract obstruction due to systolic anterior motion.

**Perspective**

The interstrut distance subtending the anterior mitral leaflet (AML) affects the nature of left ventricular outflow tract obstruction (LVOTO) due to systolic anterior motion of the AML. This model represents an effective method for the research into the prevention of LVOTO following mitral valve replacement with full preservation of the native valve.

See Commentaries on pages 259 and 261.

Left ventricular outflow tract (LVOT) obstruction (LVOTO) is an uncommon but serious complication of mitral valve surgery and transcatheter mitral valve replacement. LVOTO following surgical bioprosthetic mitral valve replacement can result from malpositioning of the prosthesis with a strut protruding into the LVOT or from systolic anterior motion (SAM) of the retained anterior mitral leaflet (AML).^1^^,^^2^ A reduction in LVOT diameter may accelerate flow sufficiently to create a Venturi effect such that the retained AML is drawn anteriorly (SAM), causing LVOTO. For this reason, the AML and its chordal attachments are usually resected at the time of bioprosthetic valve insertion. However, the valvular–ventricular interactions have an essential role in the maintenance of normal left ventricular (LV) systolic pump function, and transection of the AML chordae has been associated with deleterious effects on LV geometry and contractility.^3^, ^4^, ^5^

The objectives of this study were to develop an ovine model of LVOTO by a retained AML following bioprosthetic valve insertion and to assess the effect of interstrut distance on the predisposition to LVOTO. This model will allow research into methods to prevent LVOTO following bioprosthetic valve insertion and transcatheter mitral valve insertion when the AML is fully retained.

## Methods

### Experimental Animals

Nine healthy adult Merino first-cross sheep (mean body weight, 56.9 ± 6 kg) received humane care in accordance with the requirements of the Australian Code for the Care and Use of Laboratory Animals for Scientific Purposes 2013 (ISBN 1864965975). Animals underwent a minimum acclimatization period of 14 days, and the studies were performed at the Sydney Imaging Core Research facilities at The University of Sydney. This project was approved by the Animal Ethics Committee of The University of Sydney (Project 2017/1192). Sheep were deemed healthy (American Society of Anesthesiologists physical classification status 1) on physical examination findings before undergoing anesthesia for the procedure. Five sheep each underwent 3 valve insertions, and 4 sheep each underwent 3 sham procedures. All procedures were performed under a single anesthetic for each sheep and were terminated by euthanasia under anesthetic.

### Experimental Procedure

Sheep were premedicated intravenously with a combination of methadone (Methodyne; Jurox, Rutherford, NSW, Australia) at a dose of 0.2 mg/kg and midazolam (MIDAZolam; B. Braun, Melsungen, Germany) at a dose of 0.4 mg/kg. Anesthesia was induced by administering propofol (Propofol-Lipuro 1%; B. Braun) intravenously to effect, to facilitate orotracheal intubation. Anesthesia was maintained with inspired 1% to 2% isoflurane (Laser Isoflurane BP; PharmChem, Fort Worth, Tex) delivered in an air/oxygen mixture with a fraction of inspired oxygen between 50% and 90% and ketamine (Ketamil; Troy Laboratories, Glendenning, Australia) at an intravenous infusion rate of 20 μg/kg/minute. Morphine injections (DBL Morphine Sulfate; Pfizer, New York, NY) at 0.5 mg/kg intravenously were repeated every 4 hours. No neuromuscular blocking agents were used. Mechanical ventilation was initiated to maintain end tidal CO_2_ pressure between 35 and 50 mm Hg and oxygen saturation >93% measured using pulse oximetry. Arterial blood gas, electrolyte analysis, blood lactate levels, and activated clotting times were measured periodically throughout the anesthesia to assess physiological status and ensure normal organ perfusion. A noradrenaline infusion (Levophed; Pfizer) was administered to effect (0.1-1 μg/kg/hour intravenously) to support the maintenance of normotension.

Each sheep was placed in the right lateral recumbent position, and a left lateral thoracotomy was performed via the fifth intercostal space. The pericardium was opened, and the heart was supported in a pericardial cradle.

A transit-time ultrasonic flow probe (Transonic, Ithaca, NY) was placed around the main pulmonary artery to measure cardiac output. A straight pressure sensor–tipped catheter (Millar, Houston, Tex) was placed into the aortic arch to monitor arterial pressure. A second straight pressure sensor–tipped catheter (Millar, Houston, Tex) was inserted transapically into the LV with the pressure sensor positioned below the aortic valve. Both catheters were calibrated using the Millar Pressure Volume Loop System (MPVS) control software before cardiopulmonary bypass (CPB), and both were connected to a data acquisition hardware device (Powerlab 8/35; ADInstruments, Bella Vista, Australia) for reliable data acquisition.

All recording devices were linked to a data analysis software program (LabChart; ADInstruments), which allowed simultaneous recording of all signals. Two-dimensional epicardial and Doppler echocardiography (Vivid Q; GE Healthcare, Rydalmere, Australia) was used to rule out any cardiac anomaly before establishing CPB and to confirm the nature and severity of LVOTO when present. The LVOTO was graded as late systolic, holosystolic, or equivocal by the physician echocardiographer who was blinded to the valve orientation.

CPB was established with right atrial and aortic arch cannulation without aortic cross-clamping. Normothermia was maintained with a perfusion pressure of 70 mm Hg and a flow rate similar to the pre-CPB cardiac output. No cardioplegia was used.

**Figure 1:**
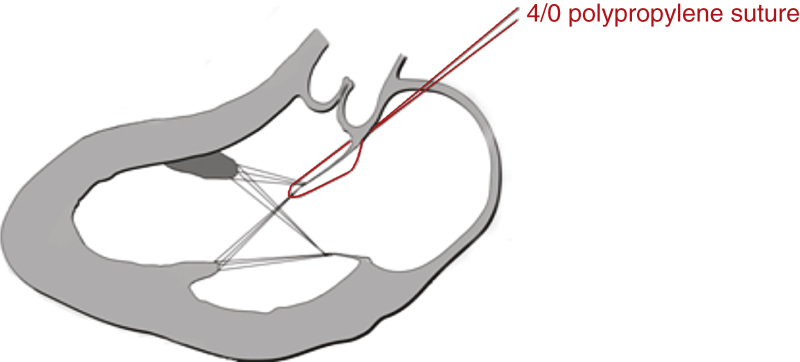
Anterior mitral leaflet technique to render the mitral valve incompetent and prevent air embolism. A 4/0 polypropylene suture is passed through the annulus at the midpoint of the base of the anterior leaflet and retrieved from the ventricular side of the leaflet. It is then passed back through the annulus such that the suture is passed entirely around the middle of the leaflet, minimizing the risk of damage to the leaflet and maximizing the effectiveness of maintaining mitral incompetence.

The empty beating heart was partially filled and a left atriotomy was performed through the base of the atrial appendage where upon the mitral valve was immediately rendered incompetent by distraction of the posterior annulus away from the AML with a pump sucker. A suture was then inserted to snare the midportion of the AML to the anterior annulus (Figure 1) to render the native valve incompetent until after left atrial closure. The LV was vented to the atmospheric pressure, and blood was evacuated using LV pump suckers. The mitral annulus was sized to ≥33 mm. The native mitral valve was fully retained.

**Figure 2:**
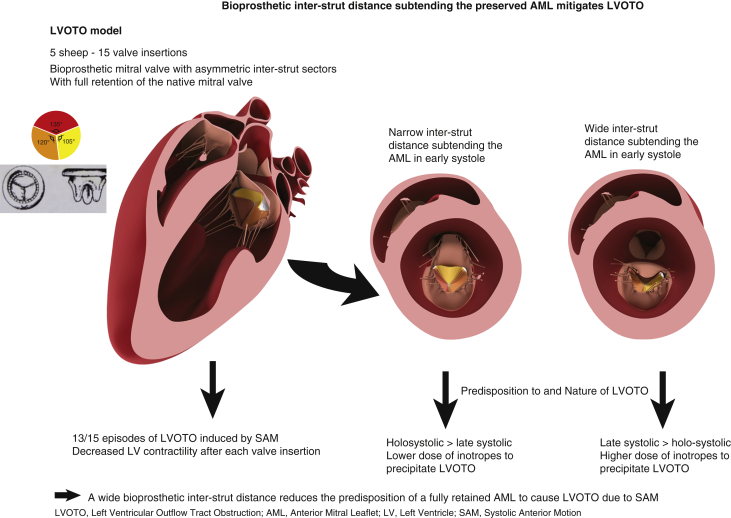
Schematic representation of a normothermic beating heart ovine model for research into the prevention of left ventricular outflow tract obstruction (*LVOTO*) using a bioprosthetic mitral valve, with full retention of the anterior mitral leaflet (*AML*). All episodes of LVOTO were due to systolic anterior motion (*SAM*). The wide interstrut distance subtending the AML was associated with a greater requirement for inotropic stress to precipitate an obstruction and was associated with late systolic rather than holosystolic obstruction.

The suture maintaining the valve incompetence was easily removed once the atrium was closed, permitting resumption of normal leaflet function. Five sheep (mean body weight, 57 ± 3.8 kg) each underwent 3 valve insertions with a 31-mm Medtronic Mosaic porcine bioprosthetic valve (Medtronic Australasia, Macquarie Park, Australia). This valve prosthesis is asymmetric with respect to the leaflet sizes, with the largest leaflet occupying a sector with an angle of 135^°^ between the supporting strut posts (Figure 2). Likewise, the angle for the smallest leaflet is 105^°^. The valve was secured to the mitral annulus with horizontal mattress sutures and positioned alternating the 105^°^ and 135^°^ interstrut angles anteriorly. For each insertion, one-third of the prosthesis circumference was secured anteriorly from commissure to commissure. The left atrium was deaired and closed, and the suture immobilizing the AML was removed. CPB was discontinued with the return of normal hemodynamic parameters. Measurements of LV and aortic pressures were recorded with simultaneous echocardiographic assessment of mitral valve function. If LVOTO did not occur spontaneously following the return of normal hemodynamic parameters, the cardiac output was increased up to twice baseline with a slow intravenous injection of adrenaline 0.1 mg.

Four sheep (mean body weight, 56.8 ± 8.8 kg) underwent a sham heart valve insertion procedure in which a bioprosthetic valve was not inserted. The left atrium was opened, and the AML was rendered incompetent. The atrium remained opened for 20 minutes, which was the average time for bioprosthetic valve insertion in the study group. Then the left atrium was deaired and closed, and the suture immobilizing the AML was removed. No adrenaline was administered to these sheep.

All procedures were performed by a senior cardiothoracic surgeon (H.S.P.) experienced in both human and ovine mitral valve surgery.

### Data Collection for Pressure Gradients

For each valve insertion or sham procedure, contractility data representing data points were collected and averaged over 2 periods of 30 seconds each immediately before any LVOTO episode or adrenaline administration. LVOTO was defined by a peak instantaneous pressure gradient >30 mm Hg. LV contractility was assessed by the dP/dt_max_ and contractility index (CI), where the CI is dP/dt_max_/Pi (Pi = instantaneous LV pressure at the time of dP/dt_max_). The CI was used to offset the effects of preload and afterload on dP/dt_max_ and to provide a better estimate of intrinsic LV contractility. When LVOTO occurred, it was resolved by resumption of CPB following echocardiographic assessment, whereupon replacement of the valve to the next rotation was performed. Therefore, the effects of LVOTO on contractility resulting from an episode of LVOTO were estimated from measurements taken after the subsequent valve insertion but before the onset of LVOTO from that insertion.

### Statistical Analysis

All calculations were performed using Stata 13 (StataCorp, College Station, Tex). The normality of dependent variables was tested using the D'Agostino *K*^2^ test. Normally distributed variables were described using mean and standard deviation. Intraclass correlation for repeated within subject measures was negligible, and thus differences in observed means were examined using a 2-sample *t* test. Nonparametric data were evaluated with the Wilcoxon rank-sum test. Repeated-measures analysis of variance was used to assess the effect of valve insertion number on measures of LV contractility. The effect of interstrut distance on the time to LVOT obstruction and the influence of LVOT duration on subsequent LV contractility were evaluated using mixed-effects linear regression. Models were fitted by backward stepwise selection and compared using the likelihood ratio test. The distribution of echocardiographic findings depending on the interstrut distance was calculated with Pearson's χ^2^ test. For all analyses, *P* < .05 was considered significant.

## Results

**Figure 3:**
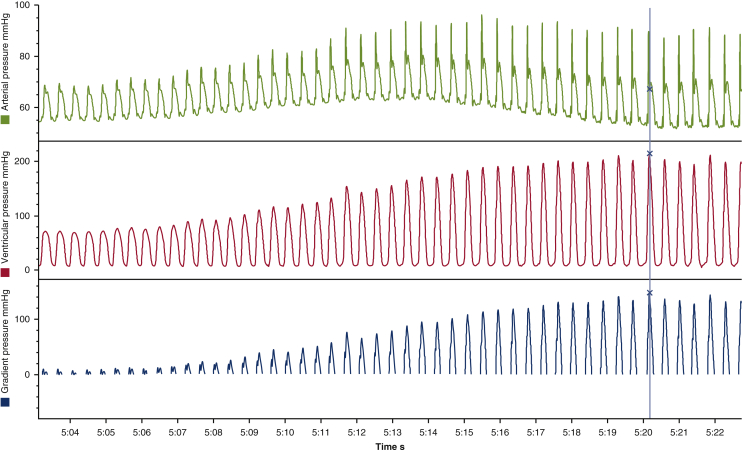
Abrupt onset of left ventricular outflow tract obstruction (LVOTO) following bioprosthetic valve insertion with full retention of the native anterior mitral leaflet. The gradient pressure (*blue trace*) is the difference between the ventricular pressure (*red trace*) and the aortic pressure (*green trace*) during systole. All pressures were recorded continuously after separation from cardiopulmonary bypass. A pressure gradient >30 mm Hg characterizes LVOTO. The markers “*X*” represent the maximal instantaneous peak gradient pressure (147.83 mm Hg) and associated ventricular pressure (214.9 mm Hg) and arterial pressure (67 mm Hg).

Thirteen of 15 valve insertions resulted in LVOTO. LVOTO was not demonstrated in the first sheep following the second and third valve insertions; therefore, 7 of 8 wide interstrut distances under the AML and 6 of 7 narrow interstrut distances resulted in LVOTO. Transapical epicardial echocardiography confirmed SAM of the AML as the cause of LVOTO in all episodes (Video 1). The onset of obstruction was abrupt, and the maximum peak instantaneous pressure gradient was 112 ± 21.2 mm Hg (range, 60-160 mm Hg) (Figure 3). The mean pressure gradient during LVOTO was 64.5 ± 13.2 mm Hg (range, 39-83 mm Hg). Increases in dP/dt_max_ and CI during LVOTO were secondary to the extreme afterload induced by the LVOTO. The dP/dt_max_ and CI increased from just before the onset of LVOTO to just after the onset by 48% and 23%, respectively.

When the wide interstrut distance subtended the AML, the time from discontinuation of CPB to the onset of LVOTO was significantly longer than when the narrow interstrut subtended the AML (8.4 ± 0.9 minutes and 1.9 ± 0.5 minutes, respectively; *P* = .0001) (Table 1). To account for potential confounders, a mixed-effects linear model was fitted, in which the fixed effect was the interstrut distance and the random effect was the subject sheep. The wide interstrut distance remained a significant predictor of increased time to LVOTO (ß = 6.5; 95% confidence interval, 4.5-8.7; *P* < .0001). LVOTO occurred spontaneously in 0 of 8 wide interstrut insertions and in 4 of 7 narrow interstrut insertions. The delay in onset of LVOTO resulted in an adrenalin bolus being given to induce LVOTO after 8 of 8 wide interstruts versus 3 of 7 narrow interstruts. The wide interstrut distance subtending the anterior leaflet was associated with a greater requirement for adrenaline boluses than the narrow interstrut distance to precipitate an obstruction (627 ± 667 μg and 108 ± 220 μg, respectively; *P* = .04) (Table 1). The sequence of valve insertion (first, second, or third insertion) did not significantly affect the predisposition to or the nature of the LVOTO.

**Table 1:** Effects of the bioprosthetic interstrut distance subtending the AML on LVOTO

Parameter	Wide interstrut distance	Narrow interstrut distance	*P* value
Time to LVOTO, min, mean ± SD	8.4 ± 0.9	1.9 ± 0.5	.0001∗
Adrenaline dose to precipitate LVOTO, μg, mean ± SD	627 ± 6.7	108 ± 220	.04†
Predominant obstruction type	Late systolic	Holosystolic	.02‡

Five sheep underwent 3 consecutive bioprosthetic valve insertions, alternating wide and narrow interstrut distances under the AML. *LVOTO*, Left ventricular outflow tract obstruction; *SD*, standard deviation.

∗ Independent-values *t* test.

† Wilcoxon rank-sum test.

‡ Pearson χ^2^ test.

When the narrow interstrut distance subtended the AML, the obstruction was classified as holosystolic on 5 of 7 occasions and late systolic on 2 of 7 occasions (Videos 1 and 2). With the wide interstrut distance, the obstruction was classified as holosystolic on 1 of 8 occasions, late systolic on 5 of 8 occasions, and equivocal on 2 of 8 occasions. The narrow interstrut distance was more often associated with a holosystolic LVOTO (*P* = .02) (Table 1, Figure 3, Videos 1 and 2). There was no difference in maximum gradients during LVOTO between holosystolic and late systolic obstructions.

**Figure 4:**
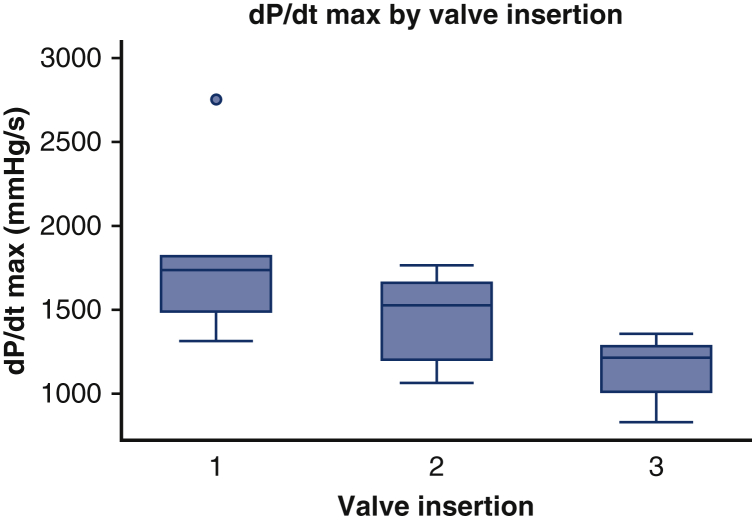
Changes in dP/dt_max_ following 3 insertions of a bioprosthetic mitral valve with full retention of the anterior mitral leaflet. dP/dt_max_ is a contractility parameter and represents the steepest slope during the upstroke of the pressure curve. Values in the *boxplots* for dP/dt_max_ (mm Hg/s) are median (interquartile range).

**Figure 5:**
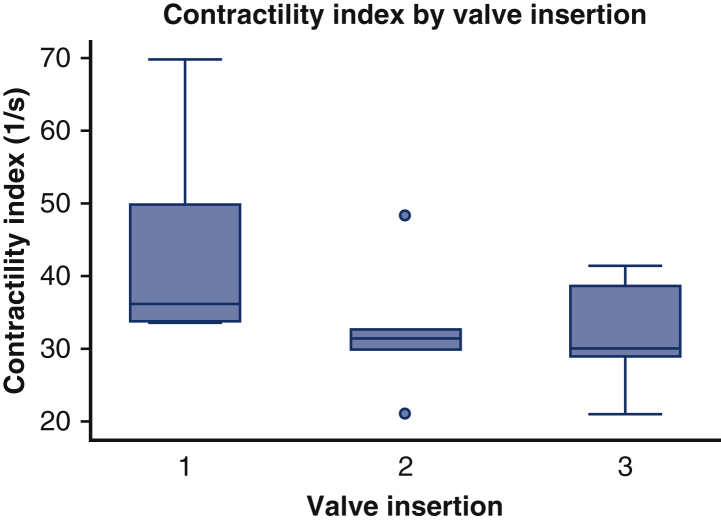
Changes in the contractility index (CI) following 3 insertions of a bioprosthetic mitral valve with full retention of the anterior mitral leaflet. The CI is dP/dtmax divided by the pressure at the time of dP/dtmax. The values in *boxplots* for the CI (1/s) are median (interquartile range).

Based on dP/dt_max_ and CI, LV contractility significantly deteriorated after each period of LVOTO (*P* = .03 and .05, respectively) (Figures 4 and 5). The duration of LVOTO (time from onset of LVOTO to resumption of CPB) significantly affected the CI measured after the subsequent valve insertion (fixed effect, duration of LVOTO; random effect, subject sheep; ß = −2.4; 95% CI, −3.2 to −1.7; *P* < .0001). However, neither the interstrut distance nor the nature of the obstruction significantly affected contractility.

There was no significant difference in the dP/dt_max_ or CI across the 3 sham surgeries. Transapical epicardial echocardiography confirmed normal mitral valve function after each sham procedure.

## Discussion

### Purpose of the Model

Resection of the anterior leaflet chordae impairs the mitral valvular ventricular interaction, resulting in reduced LV contractility.^4^^,^^5^ Mitral valve replacement for ischemic mitral incompetence has a perioperative mortality rate twice that of repair by a rigid undersized annuloplasty ring.^6^ In both repair and replacement, a rigid ring is secured to the mitral annulus. The difference is that in replacement, the prosthetic ring supports prosthetic valve leaflets and the AML is resected. It seems possible that the perioperative mortality rate from valve replacement could be reduced by retention of the AML in patients with substantially impaired LV function. However, the hazard of LVOTO associated with full retention of the AML precludes this procedure.^1^ Modification of the AML is required to prevent LVOTO, but this should be minimized to maintain the maximum preservation of LV contractility. All patients with a normally functioning AML who undergo prosthetic valve insertion are likely to benefit from preservation of LV contractility by AML retention with minimal modification sufficient to prevent LVOTO. However, valve replacement in the presence of normally functioning AML is uncommon, and this group is composed largely of patients with ischemic cardiomyopathy deemed unsuitable for valve repair. These patients are the most in need of preservation of LV contractility. This model was developed to investigate techniques to ensure avoidance of LVOTO and preservation of the valvular ventricular interaction by minimal interference with the AML at the time of bioprosthetic valve insertion.

### The Model

A beating-heart ovine model was used to avoid the adverse consequences of cardioplegic myocardial ischemia. The mean arterial blood pressure during CPB was maintained around 70 mm Hg with a flow rate of approximately 70 mL/kg/minute or a flow rate similar to the pre-bypass baseline cardiac output. Normothermia was used to minimize metabolic disturbances. This model for mitral valve research has not been reported previously.

To prevent air embolism, the mitral valve was rendered incompetent immediately after opening the left atrium. With the mitral valve incompetent and the left atrium open to atmospheric pressure, the empty LV cannot generate sufficient pressure to open the aortic valve against the pressurized aorta.

Because on-pump beating-heart surgery does not impair LV contractility, multiple open-heart interventions on CPB are possible without an extended recovery period.^7^^,^^8^ Results from the control group confirmed that there was no impairment in LV function between multiple episodes of CPB and that the deterioration in LV contractility seen after the valve insertions was most likely caused by the periods of LVOTO. LV contractility was impaired by each episode of LVOTO and by the duration of each episode. Neither the gradient during LVOTO nor the nature of LVOTO (late vs holosystolic) predicted subsequent impairment of contractility. It is possible that these findings represent relative rather than absolute contributions to impairment of contractility.

LVOTO was not induced after the second and third valve insertions in the first sheep. The LVOTO after the first valve insertion was holosystolic, and the duration of LVOTO was prolonged while echocardiographic assessment of LVOTO was performed for the first time in this project. Although the duration of LVOTO was associated with subsequent impairment of LV contractility, it was not possible to confirm that impaired LV contractility reduced the incidence of LVOTO. This is a likely limitation of the small numbers analyzed in this study.

### Prevention of LVOTO

A Medtronic Mosaic bioprosthetic valve (Medtronic, Minneapolis, Minn) was used to assess the effect of interstrut distance on the incidence of LVOTO. There was a benefit to having a wide interstrut distance subtending the AML in terms of reduced predisposition to LVOTO rather than incidence and also in terms of the nature of the LVOTO when it occurred (late systolic rather than holosystolic). The late systolic form of LVOTO that is typical in patients with hypertrophic obstructive cardiomyopathy might partly explain the variable tolerance of among patients. The late presentation with LVOTO 5 years following insertion of mitral bioprosthesis with full retention of the AML has been reported.^9^ The late systolic gradient peak in patients with hypertrophic cardiomyopathy is usually due more to myocardial narrowing of the LVOT rather than to SAM. In hypertrophic cardiomyopathy, SAM is initiated in early systole, but the LVOTO can become significant at any time during systole.^10^ The identification of SAM in these patients has been a partial indication for septal reduction therapy^11^; however, the danger of LVOTO caused by SAM of the preserved AML in any situation should not be ignored. In the first reported series of patients who underwent bioprosthetic valve insertion with a fully retained AML (n = 7), 6 patients developed LVOTO, and 5 patients were found to have SAM postoperatively. Six patients died within 2 months of surgery, and the seventh patient died after 17 months.^1^ Other cases in which valve replacement without resection of the AML-induced severe LVOTO have been reported.^2^^,^^9^^,^^12^ The prevention of LVOTO can be achieved by modification of the AML or modification of the bioprosthetic valve design. Modification of the AML to prevent LVOTO can be achieved by rectangular resection of the midportion of the leaflet with minimal resection of chordae and no other native valve modification. Transcatheter splitting of the AML has been reported in association with transcatheter mitral valve replacement for successful prevention of LVOTO.^13^ A myriad of other surgical techniques for subvalvular mitral valve apparatus preservation during mitral valve replacement to prevent LVOTO have been described,^14^ but none of these techniques has been assessed for their effect on the valvular–ventricular interaction and LV function. Modification or redesign of the bioprosthesis should involve a wide interstrut distance subtending the AML (a large anterior bioprosthetic leaflet). Reducing the profile (strut post height) is likely to reduce the risk of LVOTO, and this can be achieved on a relative basis by partial atrialization of the prosthesis during insertion or by the specific design of the prosthesis. A reduction in the anteroposterior diameter of the prosthetic valve (elliptical shape) has been shown to abolish LVOTO when present.^15^ Individual patient characteristics that predispose to LVOTO, such as aortomitral angulation, septal thickness, and a small LV cavity, should be assessed.^16^ Computed tomography software and computer-based models have been developed to predict the risk of LVOTO following transcatheter mitral valve replacement.^17^ These models may be helpful in selecting surgical patients for retention of the AML, in association with further research into the methods by which the AML can be safely preserved.

When LVOTO occurs following transcatheter mitral valve insertion, it should be determined whether SAM substantially contributes to the LVOTO or whether the obstruction is due to the prosthesis impinging in the outflow tract. There has been a suggestion in an ovine model that volume loading might aggravate the LVOTO on the basis that the mitral bioprosthesis causes a fixed diameter outflow tract, so that volume loading only increases the blood flow velocity and the tendency toward SAM.^15^ The urgency of intervention likely will be dictated by the clinical condition of the patient, but also might be as related to the early or late systolic onset of SAM as it is to the peak gradient. If surgical intervention is undertaken, partial resection of the anterior leaflet should be performed through the aortic valve via an aortotomy.^9^^,^^12^

### Limitations

Variations in creating the animal models are unavoidable, but every effort was made to reduce the variation. All procedures were performed by a senior cardiothoracic surgeon (H.S.P.) experienced in both human and ovine mitral valve surgery. Data were acquired on a small number of animals, which could have led to type II statistical errors. However, an effort was made to reuse each animal 3 times to acquire 3 sets of data per animal and reduce interindividual variability.

In this study, a bioprosthetic mitral prosthesis was used, which limits the extrapolation of our results to transcatheter mitral valve replacement, given the different design of the transcatheter valves. Assessment of holosystolic versus late systolic LVOTO was performed by a single observer and is to some extent subjective. However, this observer was blinded to the valve orientation and was experienced in ovine echocardiography. Moreover, the long-term effect of late systolic SAM and its impact of LV mechanoenergetics was not assessed in the model. Finally, none of the sheep had mitral regurgitation with attendant LV enlargement, which limits the comparison with clinical patients selected for transcatheter mitral valve replacement or mitral valve replacement.

## Conclusions

The bioprosthetic valve interstrut distance subtending the native AML influences both the predisposition to LVOTO and the systolic duration of LVOTO when it occurs. The model of bioprosthetic insertion on a normothermic beating heart presented in this study is an effective model for research into prevention of LVOTO following mitral valve replacement with preservation of the entire native mitral valve and allows multiple interventions sequentially in each animal during a single study.

### Conflict of Interest Statement

The authors reported no conflicts of interest.

The *Journal* policy requires editors and reviewers to disclose conflicts of interest and to decline handling or reviewing manuscripts for which they may have a conflict of interest. The editors and reviewers of this article have no conflicts of interest.

## Supplementary Data

Video 1 Transapical 3-chamber echocardiographic view of the holosystolic systolic anterior motion when the anterior mitral leaflet is subtended by the bioprosthetic narrow interstrut distance. The native AML (*yellow arrow*) is obstructing the left ventricular outflow tract during the entire duration of systole. *Ao*, Aorta; *LV*, left ventricle. (Scale bar: 1 cm.) Video slowed to 50% of normal speed. Video available at: https://www.jtcvs.org/article/S2666-2736(21)00111-X/fulltext.

Video 2 Transapical 3-chamber echocardiographic view of the late systolic anterior motion when the anterior mitral leaflet (AML) is subtended by the bioprosthetic wide interstrut distance. The native AML (*yellow arrow*) is closing in early systole and obstructing the left ventricular outflow tract during late systole. *Ao*, Aorta; *LV*, left ventricle. (Scale bar: 1 cm.) Video slowed to 50% of normal speed. Video available at: https://www.jtcvs.org/article/S2666-2736(21)00111-X/fulltext.
