# Supplementary figures and images for: Bioprosthetic interstrut distance subtending the preserved anterior mitral leaflet mitigates left ventricular outflow tract obstruction
Source: JTCVS Open. 2021 May 21;8:251–8. doi: 10.1016/j.xjon.2021.05.005 (PMC9390146; doi:10.1016/j.xjon.2021.05.005)

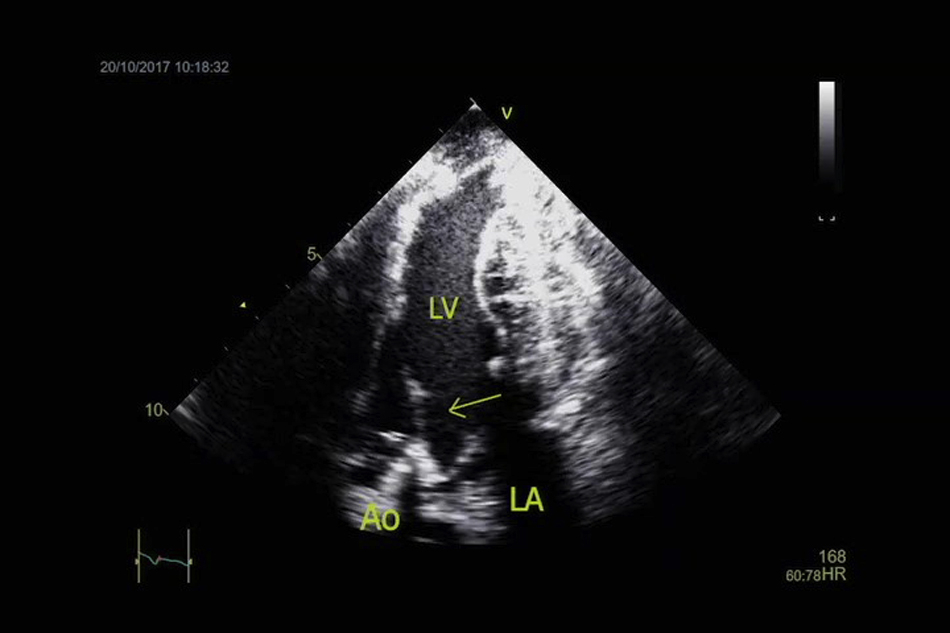

Supplement: Video 1 — Transapical 3-chamber echocardiographic view of the holosystolic systolic anterior motion when the anterior mitral leaflet is subtended by the bioprosthetic narrow interstrut distance. The native AML (yellow arrow) is obstructing the left ventricular outflow tract during the entire duration of systole. Ao, Aorta; LV, left ventricle. (Scale bar: 1 cm.) Video slowed to 50% of normal speed. Video available at: https://www.jtcvs.org/article/S2666-2736(21)00111-X/fulltext. [file fx3.jpg]

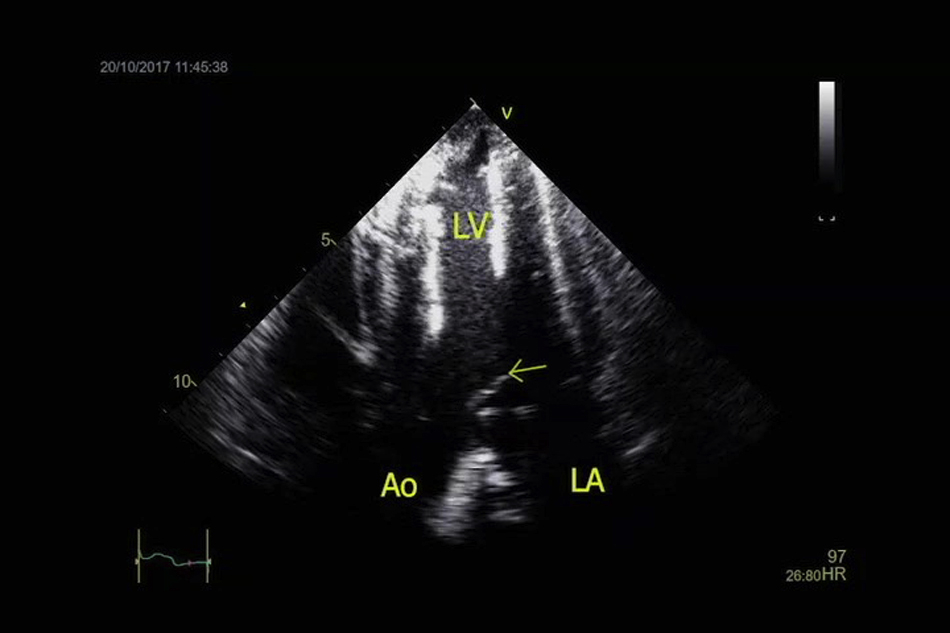

Supplement: Video 2 — Transapical 3-chamber echocardiographic view of the late systolic anterior motion when the anterior mitral leaflet (AML) is subtended by the bioprosthetic wide interstrut distance. The native AML (yellow arrow) is closing in early systole and obstructing the left ventricular outflow tract during late systole. Ao, Aorta; LV, left ventricle. (Scale bar: 1 cm.) Video slowed to 50% of normal speed. Video available at: https://www.jtcvs.org/article/S2666-2736(21)00111-X/fulltext. [file fx4.jpg]
